# 5-(4-Chloro­phen­yl)-2-fluoro­pyridine

**DOI:** 10.1107/S1600536812025378

**Published:** 2012-06-13

**Authors:** Muhammad Adeel, Fazal Elahi, M. Nawaz Tahir, Azim Khan, Peter Langer

**Affiliations:** a Department of Chemistry, Gomal University, Dera Ismail Khan, K.P.K, Pakistan; b University of Sargodha, Department of Physics, Sargodha, Pakistan; c Universität Rostock, Institut für Chemie, Abteilung für Organische Chemie, Albert-Einstein-Strasse 3a, 18059 Rostock Department of Chemistry, Germany

## Abstract

In the title compound, C_11_H_7_ClFN, the chloro­benzene and 2-fluoro­pyridine rings are oriented at a dihedral angle of 38.83 (5)°. In the crystal, there are no hydrogen-bonding interactions.

## Related literature

For a related structure, see: Elahi *et al.* (2012 ▶).
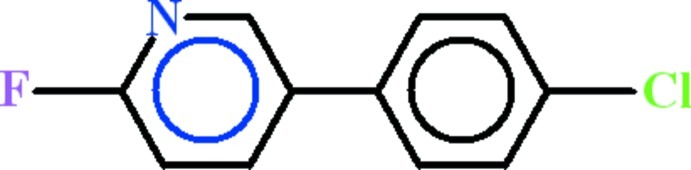


## Experimental

### 

#### Crystal data

C_11_H_7_ClFN
*M* *_r_* = 207.63
Orthorhombic, 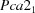

*a* = 21.1252 (14) Å
*b* = 3.8763 (3) Å
*c* = 11.7009 (8) Å
*V* = 958.16 (12) Å^3^
*Z* = 4
Mo *K*α radiation
μ = 0.37 mm^−1^
*T* = 296 K
0.26 × 0.20 × 0.18 mm

#### Data collection

Bruker Kappa APEXII CCD diffractometer
Absorption correction: multi-scan (*SADABS*; Bruker, 2005 ▶) *T*_min_ = 0.932, *T*_max_ = 0.950
4142 measured reflections
1619 independent reflections
1255 reflections with *I* > 2σ(*I*)
*R*_int_ = 0.021

#### Refinement

*R*[*F* ^2^ > 2σ(*F* ^2^)] = 0.032
*wR*(*F* ^2^) = 0.073
*S* = 1.08
1619 reflections
127 parameters
H-atom parameters constrained
Δρ_max_ = 0.11 e Å^−3^
Δρ_min_ = −0.13 e Å^−3^
Absolute structure: Flack (1983 ▶), 735 Friedel pairs
Flack parameter: −0.09 (8)

### 

Data collection: *APEX2* (Bruker, 2007 ▶); cell refinement: *SAINT* (Bruker, 2007 ▶); data reduction: *SAINT*; program(s) used to solve structure: *SHELXS97* (Sheldrick, 2008 ▶); program(s) used to refine structure: *SHELXL97* (Sheldrick, 2008 ▶); molecular graphics: *ORTEP-3 for Windows* (Farrugia, 1997 ▶) and *PLATON* (Spek, 2009 ▶); software used to prepare material for publication: *WinGX* (Farrugia, 1999 ▶) and *PLATON*.

## Supplementary Material

Crystal structure: contains datablock(s) global, I. DOI: 10.1107/S1600536812025378/bq2365sup1.cif

Structure factors: contains datablock(s) I. DOI: 10.1107/S1600536812025378/bq2365Isup2.hkl

Supplementary material file. DOI: 10.1107/S1600536812025378/bq2365Isup3.cml

Additional supplementary materials:  crystallographic information; 3D view; checkCIF report

## Figures and Tables

**Fig. 1.**
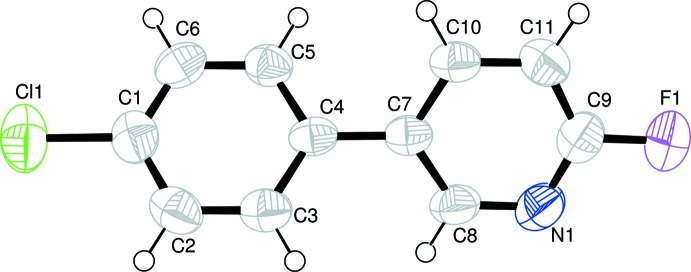
View of the title compound with the atom numbering scheme. The thermal ellipsoids are drawn at the 50% probability level.

## supplementary crystallographic information

### Comment

The title compound (I), (Fig. 1) is prepared as a precursor and for the study of biological activities.

We have reported the crystal structure of 5-(4-fluorophenyl)-2-fluoropyridine previously (Elahi *et al.*, 2012) which is related to (I). In (I) the chlorobenzene A (C1–C6/CL1) and the 2-fluoropyridine B (C7—C11/N1/F1) are planar with r.m.s. deviations of 0.0093 Å and 0.0064 Å. The dihedral angle between A/B is 38.82 (5)°. There does not exist any kind of π-interactions and the molecules must be stabilized due to van Der Wall forces.

### Experimental

To a 5 ml solution of 5-bromo-2-fluoropyridine (0.1 g, 0.568 mmol), 4-chlorophenylboronic acid (0.097 g, 0.624 mmol) in dioxane and K_3_PO_4_ (0.132 g, 0.624 mmol) was added Pd(PPh_3_)_4_ (1.5 mole %) at 373 K under N_2_ atmosphere. The reaction mixture was refluxed for 8 h. Then 20 ml of distilled water was added to the reaction mixture. The aqueous layer was extracted three times with CH_2_Cl_2_(3 × 15 ml). The organic layer was evaporated *in vacuo* and the title compound (I) was obtained as a colorless crystalline solid. Yield: 0.106 g, 91 %. M.p. 344–347 K. Crystallization from a saturated CHCl_3_ /CH_3_OH solution gave colorless crystals.

### Refinement

The H-atoms were positioned geometrically (C–H = 0.93 Å) and refined as riding with *U*_iso_(H) = *xU*_eq_(C), where *x* = 1.2 for all H-atoms.

### Crystal data

**Table:**

C_11_H_7_ClFN	*F*(000) = 424
*M**_r_* = 207.63	*D*_x_ = 1.439 Mg m^−^^3^
Orthorhombic, *P**c**a*2_1_	Mo *K*α radiation, λ = 0.71073 Å
Hall symbol: P 2c -2ac	Cell parameters from 1255 reflections
*a* = 21.1252 (14) Å	θ = 2.6–26.0°
*b* = 3.8763 (3) Å	µ = 0.37 mm^−^^1^
*c* = 11.7009 (8) Å	*T* = 296 K
*V* = 958.16 (12) Å^3^	Prism, colorless
*Z* = 4	0.26 × 0.20 × 0.18 mm

### Data collection

**Table:**

Bruker Kappa APEXII CCD diffractometer	1619 independent reflections
Radiation source: fine-focus sealed tube	1255 reflections with *I* > 2σ(*I*)
Graphite monochromator	*R*_int_ = 0.021
Detector resolution: 8.10 pixels mm^-1^	θ_max_ = 26.0°, θ_min_ = 2.6°
ω scans	*h* = −18→26
Absorption correction: multi-scan (*SADABS*; Bruker, 2005)	*k* = −3→4
*T*_min_ = 0.932, *T*_max_ = 0.950	*l* = −14→10
4142 measured reflections	

### Refinement

**Table:**

Refinement on *F*^2^	Secondary atom site location: difference Fourier map
Least-squares matrix: full	Hydrogen site location: inferred from neighbouring sites
*R*[*F*^2^ > 2σ(*F*^2^)] = 0.032	H-atom parameters constrained
*wR*(*F*^2^) = 0.073	*w* = 1/[σ^2^(*F*_o_^2^) + (0.0344*P*)^2^] where *P* = (*F*_o_^2^ + 2*F*_c_^2^)/3
*S* = 1.08	(Δ/σ)_max_ < 0.001
1619 reflections	Δρ_max_ = 0.11 e Å^−^^3^
127 parameters	Δρ_min_ = −0.13 e Å^−^^3^
0 restraints	Absolute structure: Flack (1983), 735 Friedel pairs
Primary atom site location: structure-invariant direct methods	Flack parameter: −0.09 (8)

### Special details

**Table:**

Geometry. All esds (except the esd in the dihedral angle between two l.s. planes) are estimated using the full covariance matrix. The cell esds are taken into account individually in the estimation of esds in distances, angles and torsion angles; correlations between esds in cell parameters are only used when they are defined by crystal symmetry. An approximate (isotropic) treatment of cell esds is used for estimating esds involving l.s. planes.
Refinement. Refinement of *F*^2^ against ALL reflections. The weighted *R*-factor *wR* and goodness of fit *S* are based on *F*^2^, conventional *R*-factors *R* are based on *F*, with *F* set to zero for negative *F*^2^. The threshold expression of *F*^2^ > σ(*F*^2^) is used only for calculating *R*-factors(gt) *etc*. and is not relevant to the choice of reflections for refinement. *R*-factors based on *F*^2^ are statistically about twice as large as those based on *F*, and *R*- factors based on ALL data will be even larger.

### Fractional atomic coordinates and isotropic or equivalent isotropic displacement parameters (Å^2^)

**Table:**

	*x*	*y*	*z*	*U*_iso_*/*U*_eq_	
C1	0.25579 (12)	0.5138 (6)	0.1995 (3)	0.0593 (8)	
C2	0.24833 (12)	0.3704 (7)	0.3061 (3)	0.0634 (7)	
H2	0.2834	0.3347	0.3528	0.076*	
C3	0.18888 (12)	0.2798 (7)	0.3435 (2)	0.0571 (7)	
H3	0.1840	0.1830	0.4157	0.068*	
C4	0.13552 (11)	0.3311 (6)	0.2745 (2)	0.0460 (5)	
C5	0.14486 (11)	0.4731 (6)	0.1677 (2)	0.0531 (6)	
H5	0.1102	0.5058	0.1199	0.064*	
C6	0.20444 (12)	0.5682 (6)	0.1297 (2)	0.0591 (7)	
H6	0.2096	0.6673	0.0579	0.071*	
C7	0.07137 (11)	0.2343 (6)	0.31377 (19)	0.0464 (6)	
C8	0.05152 (13)	0.2866 (7)	0.4246 (2)	0.0607 (7)	
H8	0.0804	0.3817	0.4756	0.073*	
C9	−0.04479 (14)	0.0724 (8)	0.3900 (3)	0.0646 (8)	
C10	0.02695 (11)	0.0942 (6)	0.2397 (2)	0.0521 (6)	
H10	0.0374	0.0584	0.1635	0.062*	
C11	−0.03221 (12)	0.0085 (7)	0.2783 (3)	0.0595 (7)	
H11	−0.0623	−0.0890	0.2301	0.071*	
Cl1	0.33061 (3)	0.6376 (2)	0.15406 (9)	0.0880 (3)	
F1	−0.10306 (8)	−0.0034 (5)	0.43168 (15)	0.0899 (6)	
N1	−0.00621 (11)	0.2107 (6)	0.4643 (2)	0.0696 (6)	

### Atomic displacement parameters (Å^2^)

**Table:**

	*U*^11^	*U*^22^	*U*^33^	*U*^12^	*U*^13^	*U*^23^
C1	0.0572 (16)	0.0525 (15)	0.068 (2)	0.0040 (12)	0.0070 (13)	−0.0211 (14)
C2	0.0583 (17)	0.0676 (17)	0.064 (2)	0.0100 (14)	−0.0131 (13)	−0.0165 (17)
C3	0.0652 (16)	0.0590 (16)	0.0470 (15)	0.0066 (13)	−0.0051 (12)	−0.0039 (14)
C4	0.0594 (14)	0.0404 (12)	0.0383 (14)	0.0086 (10)	−0.0039 (11)	−0.0043 (11)
C5	0.0569 (13)	0.0544 (14)	0.0479 (16)	0.0071 (11)	−0.0051 (13)	−0.0076 (15)
C6	0.0756 (18)	0.0536 (15)	0.0480 (17)	0.0056 (12)	0.0076 (13)	−0.0061 (13)
C7	0.0550 (14)	0.0421 (13)	0.0423 (16)	0.0082 (11)	−0.0046 (11)	−0.0004 (11)
C8	0.0730 (18)	0.0689 (18)	0.0402 (16)	−0.0056 (14)	−0.0050 (12)	−0.0040 (14)
C9	0.0650 (19)	0.0668 (18)	0.062 (2)	−0.0003 (14)	0.0088 (15)	0.0067 (16)
C10	0.0597 (16)	0.0539 (16)	0.0426 (15)	0.0108 (12)	−0.0054 (12)	−0.0059 (12)
C11	0.0539 (15)	0.0619 (17)	0.063 (2)	0.0036 (13)	−0.0089 (13)	−0.0048 (14)
Cl1	0.0620 (4)	0.0905 (5)	0.1114 (6)	−0.0092 (4)	0.0216 (5)	−0.0273 (6)
F1	0.0695 (10)	0.1191 (15)	0.0811 (12)	−0.0215 (10)	0.0192 (10)	0.0066 (10)
N1	0.0768 (16)	0.0824 (17)	0.0495 (15)	−0.0109 (13)	0.0091 (13)	−0.0003 (13)

### Geometric parameters (Å, º)

**Table:**

C1—C2	1.374 (4)	C6—H6	0.9300
C1—C6	1.375 (3)	C7—C8	1.378 (3)
C1—Cl1	1.735 (3)	C7—C10	1.388 (3)
C2—C3	1.376 (4)	C8—N1	1.338 (3)
C2—H2	0.9300	C8—H8	0.9300
C3—C4	1.400 (3)	C9—N1	1.306 (3)
C3—H3	0.9300	C9—F1	1.356 (3)
C4—C5	1.380 (3)	C9—C11	1.357 (4)
C4—C7	1.479 (3)	C10—C11	1.369 (3)
C5—C6	1.385 (3)	C10—H10	0.9300
C5—H5	0.9300	C11—H11	0.9300
			
C2—C1—C6	120.8 (2)	C5—C6—H6	120.5
C2—C1—Cl1	119.6 (2)	C8—C7—C10	116.1 (2)
C6—C1—Cl1	119.6 (2)	C8—C7—C4	122.2 (2)
C1—C2—C3	119.8 (2)	C10—C7—C4	121.7 (2)
C1—C2—H2	120.1	N1—C8—C7	124.9 (2)
C3—C2—H2	120.1	N1—C8—H8	117.6
C2—C3—C4	121.0 (3)	C7—C8—H8	117.6
C2—C3—H3	119.5	N1—C9—F1	114.6 (3)
C4—C3—H3	119.5	N1—C9—C11	126.4 (3)
C5—C4—C3	117.6 (2)	F1—C9—C11	119.0 (3)
C5—C4—C7	120.9 (2)	C11—C10—C7	120.4 (2)
C3—C4—C7	121.5 (2)	C11—C10—H10	119.8
C4—C5—C6	121.8 (2)	C7—C10—H10	119.8
C4—C5—H5	119.1	C9—C11—C10	116.8 (2)
C6—C5—H5	119.1	C9—C11—H11	121.6
C1—C6—C5	119.0 (3)	C10—C11—H11	121.6
C1—C6—H6	120.5	C9—N1—C8	115.3 (2)
			
C6—C1—C2—C3	0.0 (4)	C5—C4—C7—C10	37.1 (3)
Cl1—C1—C2—C3	178.4 (2)	C3—C4—C7—C10	−142.3 (2)
C1—C2—C3—C4	0.1 (4)	C10—C7—C8—N1	0.4 (4)
C2—C3—C4—C5	0.5 (4)	C4—C7—C8—N1	178.6 (2)
C2—C3—C4—C7	179.9 (2)	C8—C7—C10—C11	−1.5 (3)
C3—C4—C5—C6	−1.1 (3)	C4—C7—C10—C11	−179.7 (2)
C7—C4—C5—C6	179.5 (2)	N1—C9—C11—C10	0.7 (4)
C2—C1—C6—C5	−0.6 (4)	F1—C9—C11—C10	179.0 (2)
Cl1—C1—C6—C5	−179.00 (17)	C7—C10—C11—C9	1.0 (3)
C4—C5—C6—C1	1.2 (3)	F1—C9—N1—C8	179.9 (2)
C5—C4—C7—C8	−140.9 (3)	C11—C9—N1—C8	−1.7 (4)
C3—C4—C7—C8	39.6 (3)	C7—C8—N1—C9	1.1 (4)

## References

[bb1] Bruker (2005). *SADABS* Bruker AXS Inc., Madison, Wisconsin, USA.

[bb2] Bruker (2007). *APEX2* and *SAINT* Bruker AXS Inc., Madison, Wisconsin, USA.

[bb3] Elahi, F., Adeel, M., Tahir, M. N., Langer, P. & Ahmad, S. (2012). *Acta Cryst.* E**68**, o2070. 10.1107/S1600536812025160 PMC3393334 22807891

[bb4] Farrugia, L. J. (1997). *J. Appl. Cryst.* **30**, 565.

[bb5] Farrugia, L. J. (1999). *J. Appl. Cryst.* **32**, 837–838.

[bb6] Flack, H. D. (1983). *Acta Cryst.* A**39**, 876–881.

[bb7] Sheldrick, G. M. (2008). *Acta Cryst.* A**64**, 112–122. 10.1107/S0108767307043930 18156677

[bb8] Spek, A. L. (2009). *Acta Cryst.* D**65**, 148–155. 10.1107/S090744490804362X PMC2631630 19171970

